# Wnt/Axin1/β-Catenin Signaling Regulates Asymmetric Nodal Activation, Elaboration, and Concordance of CNS Asymmetries

**DOI:** 10.1016/j.neuron.2007.07.007

**Published:** 2007-08-02

**Authors:** Matthias Carl, Isaac H. Bianco, Baubak Bajoghli, Narges Aghaallaei, Thomas Czerny, Stephen W. Wilson

**Affiliations:** 1 Department of Anatomy and Developmental Biology, UCL, Gower Street, London WC1E 6BT, UK; 2 Institute of Animal Breeding and Genetics, University of Veterinary Medicine, Veterinarplatz 1, A-1210 Vienna, Austria; 3 University of Applied Sciences, FH-Campus Wien, Vienna Biocenter, Viehmarktgasse 2A, A-1030 Wien, Vienna, Austria

**Keywords:** DEVBIO, MOLNEURO

## Abstract

Nodal activity in the left lateral plate mesoderm (LPM) is required to activate left-sided Nodal signaling in the epithalamic region of the zebrafish forebrain. Epithalamic Nodal signaling subsequently determines the laterality of neuroanatomical asymmetries. We show that overactivation of Wnt/Axin1/β-catenin signaling during late gastrulation leads to bilateral epithalamic expression of Nodal pathway genes independently of LPM Nodal signaling. This is consistent with a model whereby epithalamic Nodal signaling is normally bilaterally repressed, with Nodal signaling from the LPM unilaterally alleviating repression. We suggest that Wnt signaling regulates the establishment of the bilateral repression. We identify a second role for the Wnt pathway in the left/right regulation of LPM Nodal pathway gene expression, and finally, we show that at later stages Axin1 is required for the elaboration of concordant neuroanatomical asymmetries.

## Introduction

Structural and functional asymmetries are common features of the nervous systems of both invertebrates and vertebrates (Halpern et al., 2003; Hobert et al., 2002). The best described neuroanatomical asymmetries in vertebrates are found in the diencephalic epithalamus, where both the habenulae and the dorsally adjacent pineal complex are lateralized in many species. The epithalamus is part of a conserved output pathway of the limbic system, connecting telencephalic nuclei to the interpeduncular nucleus (IPN) in the ventral midbrain (Sutherland, 1982).

During early development in zebrafish, bilaterally located parapineal cells migrate leftward from the pineal complex to form a left-sided nucleus that sends ipsilateral axonal projections to the left habenula (Concha et al., 2003). The paired habenular nuclei themselves show various asymmetries, including differences in gene expression, subnuclear regionalization, timing of neuronal differentiation, and neuropil organization (Aizawa et al., 2005, 2007; Concha and Wilson, 2001; Gamse et al., 2003, 2005). Left-right asymmetries in habenular neuronal organization are converted into a dorsal-ventral asymmetry in the targeting of the habenular axons in the midbrain IPN, with left-sided habenular axons predominantly innervating the dorsal IPN and right-sided axons projecting to the ventral IPN (Aizawa et al., 2005).

The parapineal influences the elaboration of habenular asymmetries. For instance, the parapineal modulates gene expression in the left habenula, and ablation of parapineal cells results in the left habenula adopting some right-sided character (Concha et al., 2003; Gamse et al., 2003, 2005; Kuan et al., 2007). On the other hand, ablation of left-sided habenula precursors can influence the orientation of parapineal migration (Concha et al., 2003). Taken together, these results suggest that there is communication between the various structures in the epithalamus to ensure coordinated and consistent elaboration of lateralized neuroanatomical asymmetries.

The earliest known indication of brain asymmetry in zebrafish is the expression of Nodal pathway genes within the left epithalamus from about 18 hpf (Halpern et al., 2003). Epithalamic Nodal signaling influences the laterality of the habenulae and parapineal, but asymmetry per se appears to be established independently of this pathway (Concha et al., 2000, 2003; Liang et al., 2000). As the Nodal pathway is activated unilaterally in the epitahalmus, other mechanisms must act upstream to initiate this asymmetry. Within the lateral plate mesoderm (LPM), Nodal signaling has evolutionarily conserved roles in the development of asymmetries (Hamada et al., 2002), and in zebrafish, it appears that activation of Nodal pathway genes in the left epithalamus is dependent upon the activity of the Nodal ligand Southpaw (Spw) emanating from the left LPM (Long et al., 2003). Whether this activity of Spw is direct or indirect is unknown. We have previously proposed that the role of left-sided LPM Nodal signaling may be indirect, through removal of repression of Nodal pathway gene expression in the left epithalamus (Concha et al., 2000).

In this study, we address the role of the Wnt/Axin1/β-catenin signaling pathway in the regulation of asymmetric Nodal pathway gene expression and in the elaboration of brain asymmetries. The role of this pathway in the development of brain asymmetries has not previously been assessed, but some studies suggest that Wnt signaling can influence visceral asymmetries. For instance, overexpression of Xwnt8 in *Xenopus* can lead to cardiac left-right reversals (Danos and Yost, 1995; Nascone and Mercola, 1997) as can overactivation of the Wnt/β-catenin pathway in medaka (Bajoghli et al., 2007). In chick, Wnt/β-catenin signaling is suggested to be a left determinant of Nodal pathway gene expression in the LPM, as early upregulation of the pathway results in bilateral Nodal gene expression (Rodriguez-Esteban et al., 2001). Furthermore, mice lacking Wnt3a exhibit asymmetry defects that are likely due to a requirement for Wnt3a acting in and around the node during the period when asymmetries first become evident (Nakaya et al., 2005).

Here, we use a variety of approaches to establish roles for Wnt/β-catenin signaling and the Wnt pathway scaffolding protein Axin1 in both the regulation of Nodal pathway activation and in the differentiation of lateralized brain nuclei. *masterblind* (*mbl*) embryos carry a mutation in Axin1 that disrupts the binding of GSK3β, reducing the ability of GSK3β to degrade β-catenin and consequently leading to overactivation of Wnt/β-catenin signaling in the anterior neural plate (Heisenberg et al., 1996, 2001; Houart et al., 2002; Masai et al., 1997; Van de Water et al., 2001). We find that *mbl* mutant embryos show bilateral activation of Nodal pathway genes in the epithalamus but not the viscera. This activation can occur independently of the activity of Spw, suggesting that overactivation of Wnt signaling bilaterally removes repression of epithalamic Nodal pathway gene expression. We provide evidence that this likely reflects a role for Wnt signaling during late gastrulation. Later overactivation of Wnt signaling during somitogenesis stages can disrupt lateralized Nodal pathway gene expression concordantly in the LPM and brain in both zebrafish and medaka. This is consistent with a role for Spw in the ipsilateral removal of repression of epithalamic Nodal pathway gene expression. Finally, we show that Axin1 is also required downstream of Nodal signaling during the elaboration of epithalamic asymmetries. Our results provide evidence that the Wnt/Axin1/β-catenin signaling pathway plays several critical roles during the establishment and elaboration of asymmetries in the forming CNS.

## Results

### Alterations to Wnt Signaling Early in Development Disrupt the Asymmetry of Nodal Gene Expression

Previous studies have shown that Wnt signaling influences early development of the forebrain (reviewed in Wilson and Houart, 2004), but none have examined the influence of this pathway on CNS asymmetries. As a first approach to address this issue, we manipulated Wnt signaling early in development. Activation of the Wnt pathway through early injection of RNA encoding Wnt8b disrupts the left-sided activation of Nodal pathway genes in the body and brain (see Figure S1 in the Supplemental Data available with this article online). As these experiments do not reveal the time or place at which the Wnt pathway can influence asymmetry, we next analyzed *mbl* embryos that carry a mutation in the GSK3β binding domain of Axin1, resulting in enhanced Wnt activity in the anterior neural plate during late gastrulation (Heisenberg et al., 2001; Houart et al., 2002; Van de Water et al., 2001), and designed other experiments that allowed us to temporally manipulate levels of Wnt signaling.

### Axin1 Is Required to Restrict Nodal Pathway Gene Expression to the Left Side of the Epithalamus

**Figure 1:**
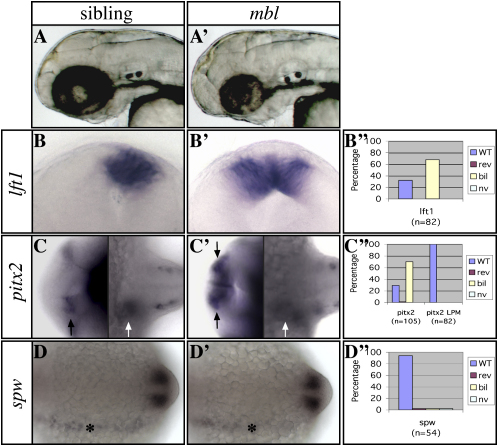
The *mbl* Mutation in *axin1* Causes Brain-Specific Loss of Asymmetric Nodal Pathway Gene Expression (A and A′) Lateral views of the head with anterior to the left of 2-day-old living wild-type (left) and *mbl* mutant (right) embryos. (B and B′) Frontal views of the epithalamus (dorsal to the top) of 24s stage wild-type and *mbl* embryos. (C and C′) Dorsal views of the brain (left) and trunk LPM (right) of 24s stage wild-type and *mbl* embryos with anterior to the left. (D and D′) Dorsal views of the trunk LPM of 14s stage wild-type and *mbl* embryos with anterior to the left. The markers used to assess asymmetries are indicated to the left of the panels. (B″–D″) Graphs illustrate the percentage of *mbl* embryos with wild-type (WT) left, reversed (rev) right, bilateral (bil), or not visible (nv) Nodal pathway gene expression (see also Table S1). Note the loss of asymmetry in Nodal pathway gene expression in the epithalamus ([B], [B′], and black arrows in [C] and [C′]), but not the lateral plate mesoderm (LPM; white arrows in [C] and [C′] and asterisks in [D] and [D′]) in mutants. The expression analysis of *lft1* and *pitx2* in all figures refers to expression in the brain, unless indicated otherwise.

*mbl* embryos show a CNS phenotype of variable, background-dependent, expressivity (Heisenberg et al., 2001; Sanders and Whitlock, 2003; Van de Water et al., 2001), in which eyes and telencephalon are reduced and posterior neural structures are expanded. For our analyses, we used a line of fish in which homozygous *mbl^tm213^* mutants showed a clearly distinguishable but mild phenotype, in which brain regionalization and epithalamic size were relatively normal (Figure 1A and Experimental Procedures).

In contrast to their normally left-sided expression in wild-type embryos or *mbl*/+ siblings, the Nodal pathway genes *lft1*, *pitx2*, and *cyc* are expressed bilaterally in the epithalamus of about 70% of *mbl* embryos (Figures 1B and 1C and Table S1). However, within the LPM, expression of *pitx2* and later markers of liver, pancreas, and heart are unaffected (Figure 1C and Table S1). These results suggest that altered Wnt/Axin1/β-catenin pathway activity can affect asymmetric epithalamic gene expression independently of any effects on lateralized Nodal gene expression in the LPM.

### The *mbl* Mutation Can Activate Epithalamic Nodal Signaling Independent of Spw Activity

The Nodal ligand Spw is expressed in the left LPM but not in the brain of both wild-type and *mbl* mutant embryos (n = 51/54; Table S1 and Figure 1D). However, it is required to activate Nodal pathway genes in the left epithalamus, either directly (Long et al., 2003) or indirectly, for instance, through ipsilateral alleviation of repression of epithalamic Nodal pathway gene expression (Concha et al., 2000). If the former is correct, then removal of Spw activity in *mbl* embryos should result in the loss of epithalamic Nodal pathway activation. However, if repression of epithalamic Nodal expression is lost in *mbl* embryos, then Nodal activation should occur independently of Spw activity. To address this issue, we abrogated Spw activity in *mbl* and sibling embryos.

**Figure 2:**
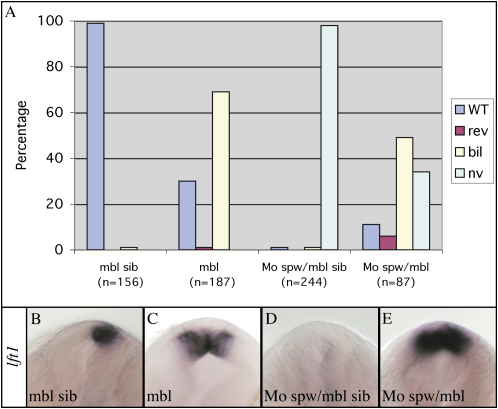
The *mbl* Mutation Can Activate Epithalamic Nodal Pathway Genes Epistatic to Loss of Spw Activity (A) The graph illustrates the percentage of embryos with wild-type (WT) left, reversed (rev) right, bilateral (bil), or not visible (nv) Nodal pathway gene expression in the epithalamus of wild-type (mbl sib) *mbl* (*mbl*^tm213/tm213^), *spw* morphant (MoSpw/mbl sib), and *mbl;spw* morphant (MoSpw/mbl) embryos. The knockdown of Spw function results in the absence of *lft1* and *pitx2* expression only in the presence of wild-type Axin1. (B–E) Frontal views of the epithalamus (dorsal to the top) of 24s stage wild-type (mbl sib) *mbl*, *spw* morphant (MoSpw/mbl sib), and *mbl;spw* morphant (MoSpw/mbl) embryos analyzed for *lft1* expression.

Although Spw activity is required to activate epithalamic Nodal signaling in wild-type embryos, this is not the case in the majority of *mbl* embryos (Figure 2A). Thus, in *spw* morphants, *pitx2* and *lft1* expression in the brain was absent (n = 163/166 and n = 77/78, respectively; Figures 2B and 2D), whereas *spw* morphant *mbl* mutants showed *pitx2* (n = 32 of 48) and *lft1* (n = 25 of 39) expression (Figures 2C and 2E). Thus, the alterations in Wnt/Axin1/β-catenin pathway activity are largely epistatic to the loss of Spw.

These results show that abrogation of Axin1 activity can lead to activation of Nodal pathway gene expression in the epithalamus independently of the activity of Spw. This is consistent with the idea that enhanced Wnt signaling in the neural plate alleviates repression of epithalamic Nodal gene expression, removing the requirement for Spw activity.

### Activation of Wnt Signaling at Mid-Somite Stages Concordantly Disrupts Nodal Pathway Gene Expression in Both LPM and Epithalamus

Although *mbl* mutants show no asymmetry defects in the LPM, other manipulations suggested that disruptions to Wnt pathway activity could disturb asymmetric Nodal pathway gene expression in both LPM and brain (Figure S1 and Table S1). To address the possibility that Wnt signaling may be able to influence LPM asymmetries during the period when they are being established, we temporally manipulated Wnt signaling through bathing embryos in lithium chloride (LiCl) to suppress GSK3β function and thereby activate the Wnt/β-catenin pathway (Stambolic et al., 1996). While LiCl treatments at blastula/gastrula stages result in posteriorization of the brain (Macdonald et al., 1994), treatments at later stages do not obviously alter CNS morphology (Kim et al., 2002) (data not shown). We treated embryos with LiCl for 15–20 min periods between 80% epiboly and 22 somites (Experimental Procedures) and subsequently assessed Nodal pathway gene expression and body and brain asymmetries.

**Figure 3:**
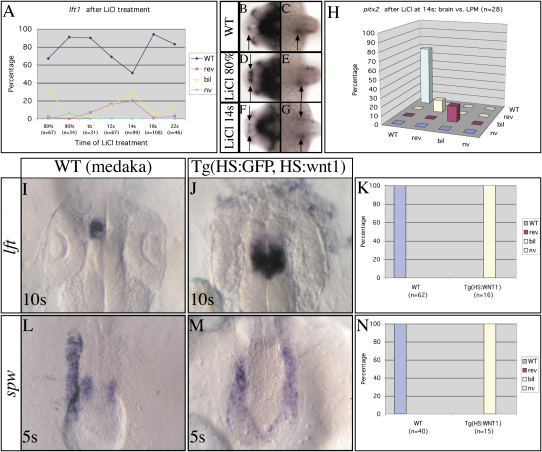
Manipulating Wnt Signaling during Mid-Somite Stages Disrupts the Laterality of Nodal Pathway Expression in Both LPM and Brain (A, H, K, and N) Graphs illustrate the percentage of embryos with wild-type (WT) left, reversed (rev) right, bilateral (bil), or not visible (nv) Nodal pathway gene expression (see also Tables S2 and S3). (A) Zebrafish embryos were treated with lithium chloride (LiCl) at the stages indicated and analyzed for Nodal pathway gene expression in the brain (and LPM in [H]) at 24s stage (see also Experimental Procedures). (B–G) Dorsal views of the (B, D, and F) brain and (C, E, and G) trunk LPM of 24s stage wild-type and *mbl* embryos, with anterior to the left analyzed for *pitx2* expression. The arrows indicate the sites of *pitx2* expression (epithalamus in [B], [D], and [F]; LPM in [C], [E], and [G]). (B–E) LiCl treatment of embryos at 80% epiboly results in the loss of *pitx2* asymmetry in the brain alone, whereas (F–H) treatments at 14 somite stage result in disruption of expression concordantly in brain and LPM. (I, J, L, and M) Dorsal views of medaka embryos (anterior to the top, stages are indicated at the bottom left of each panel) showing (I and J) *lft* expression in the epithalamus and (L and M) *spw* expression in the LPM. Heat shock treatments were performed at 4s stage (I and J) and at 2s stage (L and M). Heat shock activation of (J and M) *wnt1* in Tg(HS:GFP, HS:wnt1) transgenic medaka embryos causes bilateral Nodal pathway gene expression in the CNS and LPM (K and N). (K and N) Heat shock of wild-type embryos had no effect on *lft* or *spw* expression.

Asymmetric expression of Nodal pathway genes in the epithalamus, but not the LPM, is disrupted by LiCl treatment at 80% epiboly. About a quarter of embryos treated with LiCl at this stage showed bilateral expression of *lft1* or *pitx2* in the epithalamus at 24 somites (n = 204; Figures 3A–3E and Table S2), but none showed bilateral *pitx2* expression in the LPM (n = 52). This phenocopies *mbl* mutants, lending support to the conclusion that overactivation of Wnt signaling in the anterior neural plate during late gastrulation removes repression of epithalamic Nodal pathway gene expression. At no other stage from late gastrula until 22 somite stage did LiCl treatment affect epithalamic Nodal asymmetries without concomitantly affecting gene expression in the LPM (Table S2).

In addition to the early phase of sensitivity to LiCl treatment, many embryos treated between 12 and 14 somites showed concordant, predominantly bilateral, Nodal pathway gene expression in the brain and LPM (Figures 3A and 3F–3H and Table S2 and S3). As expected from the disruption to Nodal pathway activity, embryos subsequently showed disrupted laterality of CNS and visceral asymmetries (Table S3). This result suggests that decreased GSK3β activity (and enhanced Wnt activity) during a narrow time window at mid-somitogenesis stages disrupts the restriction of Nodal pathway gene expression to the left side of the embryo.

The laterality of expression of Nodal pathway genes in the brain was concordant with expression in the LPM in these experiments, suggesting that by mid-somite stage, the activation of Nodal signaling in the brain is dependent upon Nodal signaling emanating from the LPM. To test this, we treated *spw* morphants with LiCl at 14s and found no expression of *pitx2* (n = 32 of 32) or *lft1* (n = 47 of 47). Thus, by mid-somite stages, activation of epithalamic Nodal signaling is dependent upon LPM Nodal activity.

### A Role for Wnt/Axin1/β-Catenin Signaling in Regulating Asymmetry during Early Somitogenesis Is Conserved among Teleosts

To further validate our conclusions suggesting a temporally restricted role for Wnt signaling and to assess whether such a role is conserved across species, we performed comparable experiments in the distantly related teleost, medaka (Wittbrodt et al., 2002). Asymmetries of gene expression and neuroanatomy in the brain of medaka are similar to those in zebrafish (M.C. and F. Loosli, unpublished data; Soroldoni et al., 2006). As in zebrafish, the parapineal is left-sided, the bilateral habenulae have asymmetries in neuropil organization and show dorsoventral segregation of left and right axon terminals in the target IPN nucleus (Figure S2). At earlier stages, Nodal pathway genes are expressed asymmetrically in the LPM and epithalamus similar to zebrafish (Figures 3I and 3L) (Bajoghli et al., 2007; Soroldoni et al., 2006). These observations suggest that the regulatory mechanisms underlying the formation of brain asymmetries are likely to be comparable between medaka and zebrafish. However, in medaka, CNS development is advanced with respect to somitogenesis compared to zebrafish, and so, for instance, Nodal pathway genes are activated in the epithalamus at “earlier” somite stages (Soroldoni et al., 2006).

Ubiquitous activation of *wnt1* in a Tg(HS:GFP, HS:wnt1) line at 2–4 somite stage results in bilateral Nodal pathway gene expression in the epithalamus and LPM (Figures 3J, 3K, 3M, and 3N). These data indicate that the activity of the Wnt signaling pathway in the regulation of asymmetry of Nodal pathway gene expression is evolutionarily conserved, at least among teleosts.

### Axin1 Regulates the Elaboration of Epithalamic Asymmetries Downstream of Epithalamic Nodal Pathway Activity

Previous studies have shown that disrupting unilateral left-sided epithalamic Nodal pathway gene expression disrupts the laterality of neuroanatomical asymmetries but does not affect the development of the asymmetries per se (Aizawa et al., 2005; Concha et al., 2000, 2003; Halpern et al., 2003). Consistent with this, the various manipulations that resulted in altered Nodal signaling in the LPM and epithalamus resulted in disrupted laterality of visceral and brain structures (Figure S1 and Table S3). However, as described in the sections below, we find that *mbl* mutants exhibit several additional phenotypic defects in lateralized epithalamic structures that cannot be explained as a consequence of disrupted Nodal signaling.

### The Migration of Parapineal Cells Is Delayed in *mbl* Mutants

**Figure 4:**
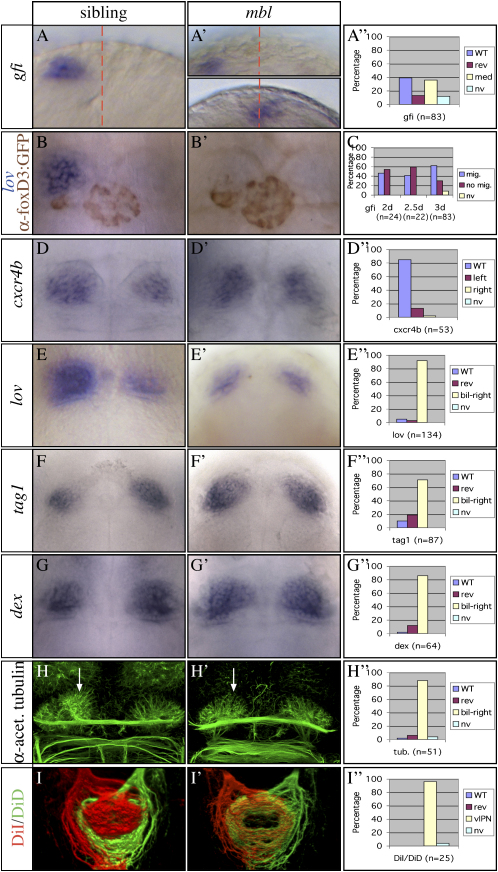
*mbl* Mutants Have Parapineal Migration Defects and Bilaterally Symmetric Habenulae (A–H and A′–H′) Dorsal or frontal views of the epithalamus (anterior to the top) of (A, A′, D, and D′) 2-day-, (B and B′) 2.5-day-, (F and F′) 3-day-, and (E, E′, G, G′, H, and H′) 4-day-old wild-type and *mbl* mutant embryos. All markers used in the panels are indicated on the left (the text color matches the expression domain color in double labelings). (A–A″ and C) *gfi* is expressed exclusively in the parapineal (midline is indicated by the red dotted line). Of the two *mbl* embryos shown, one shows normally migrated parapineal cells, and the other shows parapineal cells at the midline. (B and B′) Embryos carried a foxD3:GFP transgene [mbl^tm213^ × Tg(foxD3:GFP)]. Double labeling shows that, even in the presence of migrating parapineal cells, *lov* gene expression remains low in the left habenula of the mutant (B′). (H and H′) The arrows mark the neuropil of the medial left habenula, which is reduced in the *mbl* mutant. (I and I′) Dorsal views of 3D reconstructions of confocal images of habenular axon terminals in the target IPN nucleus labeled with lipophilic dyes as indicated. (A″ and D″–H″) Graphs illustrate the percentage of embryos with wild-type (WT) left, reversed (rev) right, medial (med), bilateral (bil), or not visible (nv) gene expression or neuropil formation. Bilateral right (bil-right) indicates that both habenulae exhibit the profile of gene expression or neuropil formation characteristic for the right habenula of wild-type embryos. The graph in (I″) shows that in nearly all *mbl* embryos the axonal projections coming from the habenulae intermingle in the ventral IPN.

Analysis of *mbl* mutant brains showed that Axin1 activity is required for the correctly timed migration of cells that contribute to the left-sided parapineal nucleus. In wild-type embryos, parapineal cells are initially located on both sides of the epithalamic midline, but by about 28 hpf they initiate a leftward migration (Concha et al., 2003). In contrast, only about half of 2 day *mbl* embryos showed clearly migrated parapineal cells (Figures 4A and 4C and Table S1). Expression of the parapineal-specific marker *gfi* (Dufourcq et al., 2004) in about 90% of mutant embryos confirmed that this was a migration deficit rather than a failure to specify parapineal cells. Between 2 and 3 days of development, *mbl* mutants with migrated parapineal cells increased by more than 20% (*gfi* expression: 62%, n = 83; Figure 4C), and by 4 days, almost all *mbl* embryos analyzed had a migrated parapineal (foxd3:GFP transgene, data not shown). These data indicate that Axin1 is required for the correct onset of parapineal cell migration.

Assessment of the position of the parapineal in *mbl* embryos showed that laterality is disrupted but is not fully randomized as is the case in other mutants with bilateral epithalamic Nodal pathway gene expression (Concha et al., 2000). Thus, when a lateralized parapineal was present at 2–2.5 dpf, it was mostly on the left (81.3% left-sided, 18.7% right-sided, n = 91, assessed by *gfi* and *otx5* expression). Even accounting for the variable Nodal expression phenotype of *mbl* embryos, which predicts 64% left-sided parapineal cells, the greater leftward bias in parapineal cell migration in *mbl* embryos is statistically significant (p < 0.001, binomial test). To verify this result, we generated a double transgenic *mbl* mutant line, in which GFP is expressed under the control of both the *lft1* promoter (a reporter for Nodal signaling [Aizawa et al., 2005]) and the *foxD3* promoter (to label the parapineal [Concha et al., 2003; Gilmour et al., 2002]). Analysis of *mbl* mutants that exhibited bilateral *lft1*:GFP expression at 20 hpf (n = 52) showed 35% with parapineal migration at 2 days, two-thirds of which were positioned to the left and one-third to the right. Together with our analysis of *gfi* and *otx5* expression (Table S1), these data suggest that there is still a left-sided bias in Nodal signaling in *mbl* embryos with apparently bilateral epithalamic Nodal pathway gene activation and/or that the direction of parapineal migration is influenced by factors other than the laterality of Nodal pathway gene expression in mutants.

### *mbl* Embryos Develop Largely Symmetric Habenulae with Right-Sided Character

Although the parapineal is eventually lateralized, the habenulae of *mbl* embryos develop symmetrically. Expression of the superficially symmetrical habenular markers *cxcr4b* (n = 45/53) and *zic2a* (n = 15/15) is not obviously affected in *mbl* mutants (Figure 4D and data not shown). However, habenular genes that are asymmetrically expressed in wild-type embryos exhibit largely symmetric expression in *mbl* mutants (Figures 4E–4G and Table S1). The enhanced left-sided expression of *lov* (Gamse et al., 2003) is reduced to right-sided expression levels in mutants (Figure 4E), whereas the expression of *dex* (Gamse et al., 2005) and *tag1* are increased in the left-habenula to right-sided levels (Figures 4F and 4G). Consistent with a double right-sided phenotype, the habenular neuropil of *mbl* embryos is symmetrical, exhibiting a reduction of the more complex medial labeling normally seen in the left habenula (Figure 4H and Table S1).

Assessment of the projection patterns of left- and right-sided habenular neurons further supports the conclusion that both habenulae have largely right-sided character in *mbl* mutants. Whereas the left habenula predominantly innervates the dorsal IPN in wild-type embryos (Figure 4I; Aizawa et al., 2005), left habenular axon terminals were exclusively localized to the ventral IPN in *mbl* mutants, where they intermingled with right habenula axons (Figure 4I′). Thus, gene expression, neuropil labeling, and habenular axon targeting all show that the disruption of Axin1 function in *mbl* mutants results in the formation of two habenulae with right-sided character.

Given that the parapineal confers some aspects of left-sided character to left-sided habenular neurons in wild-type embryos (Concha et al., 2003; Gamse et al., 2003, 2005; Kuan et al., 2007), it is surprising that about 90% of *mbl* embryos show double-right habenulae despite the presence of a parapineal in nearly all mutants, which migrates at the correct time in about 50% of mutants (Figures 4A–4C and Table S1). This observation implies that, unlike in wild-type embryos, the parapineal in *mbl* embryos does not confer left-sided character to habenular cells or that the habenular cells are not able to respond to signals from the parapineal.

### Epithalamic Asymmetries Are Largely Uncoupled in *mbl* Embryos

**Figure 5:**
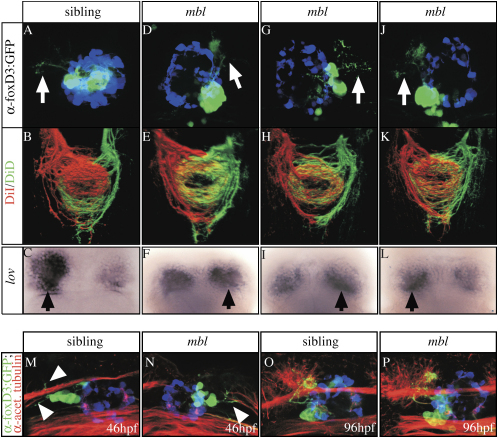
Epithalamic Asymmetries Are Largely Uncoupled in *mbl* Embryos (A–P) Dorsal views of (A–L) 4-day-old embryos derived from a *mbl^tm213^* × Tg(foxD3:GFP);Tg(lft1:GFP) incross were analyzed for (A, D, G, and J) parapineal migration and projections. The labeling performed and the genotype of the embryos analyzed are indicated on the left and at the top, respectively. (A, D, G, J, M, and N) White arrows mark parapineal projections toward the left or right habenula; pineal cells are pseudocolored in blue. (B, E, H, and K) Axonal projections into the IPN and (C, F, I, and L) *lov* gene expression (overdeveloped, black arrows mark the side of slightly more intense *lov* gene expression). The habenulae of all *mbl* embryos exhibit the projection pattern characteristic for the right habenula, irrrespective of parapineal projections. (M–P) The onset ([M and N]; arrowheads) and targeting (O and P) of parapineal projections is superficially normal in the *mbl* embryos, irrespective of the migration of parapineal cells. The pineal cells are pseudocolored blue.

To determine the relationship between the various epithalamic asymmetries in individual *mbl* mutants, we analyzed Nodal pathway activation, parapineal migration, parapineal neuron projections, gene expression in the habenulae, and projections from the habenulae to the IPN in mutants carrying *lft1*:GFP and *foxD3*:GFP transgenes (Aizawa et al., 2005 and Figures 5A, 5D, 5G, and 5J). Of 52 mutant embryos that showed bilateral *lft1*:GFP expression in the dorsal diencephalon at 20 hpf, about 65% showed no migration of parapineal cells at 48 hpf. Twelve embryos with either migrated (n = 3 left and 3 right) or nonmigrated parapineal cells (n = 6) were selected for further analysis. At 4 days of development, all 12 of these embryos exhibited a migrated parapineal. Irrespective of whether parapineal cells showed normal or delayed migration, parapineal neurons projected axons to one or the other habenula, or in one case to both habenulae (Figures 5D, 5G, and 5J and data not shown). Despite innervation by parapineal axons, habenular efferent axons always predominantly innervated the ventral IPN, suggesting double right-sided habenular character (n = 12/12; Figures 5E, 5H, and 5K). Subsequent analysis showed reduced, double right-sided *lov* gene expression (data not shown, see Figure 4E′ for reduced *lov* expression). However, to look for subtle differences in *lov* expression, we incubated the brains in the color reaction substrate for a further 24 hr. Following this procedure, we observed slightly higher levels of *lov* expression in the habenula innervated by parapineal projections. This suggests that, in *mbl* embryos, there is still residual communication between the parapineal and habenular cells.

### Parapineal Neurons Innervate the Habenulae in *mbl* Embryos

The mechanism by which the parapineal influences lateralized gene expression in the habenulae is not understood. One possibility is that the parapineal axons that innervate habenular neurons influence gene expression and efferent connectivity of these neurons. We speculated that if this is the case, then delayed innervation of the habenulae in *mbl* embryos may compromise the ability of parapineal axons to influence habenular neuron connectivity.

To address this issue, we analyzed embryos at the time when parapineal neurons first send projections toward the habenula (between 40 and 50 hpf) and found that migrated and nonmigrated parapineal neurons in *mbl* embryos have projections by 46 hpf comparable to wild-types (Figures 5M and 5N). Thus, parapineal neurons can project axons even if somal migration is disrupted. Furthermore, it is unlikely that mistargeting of *mbl* parapineal axons underlies the failure of the parapineal to influence habenular laterality, as the axons appear to terminate in the same medial region of the habenular neuropil (although neuropil is reduced) as in wild-type embryos (Figures 5O and 5P). These data indicate that Axin1 activity has no obvious effect upon parapineal neuron axogenesis and targeting but is important for the communication from parapineal cells that influences habenular laterality, most likely acting in either the parapineal, the habenulae, or both.

## Discussion

In this study, we show that Wnt/Axin1/β-catenin signaling is involved reiteratively during the establishment and concordant elaboration of brain and body asymmetries. Activation of Wnt signaling during late gastrula stages abrogates asymmetry of expression of Nodal pathway genes in the brain but not the LPM, and we suggest that this occurs through the loss of bilateral repression of Nodal pathway activation in the prospective epithalamus. Widespread activation of the pathway during mid-somite stages results in concordant disruption to asymmetric Nodal pathway gene expression in both the LPM and brain, suggesting that by this stage Nodal activation in the brain is dependent upon Nodal signaling in the LPM. Downstream of Nodal pathway activity in the epithalamus, Axin1 is required both for development and concordance of parapineal and habenular asymmetries.

### Enhanced Wnt Signaling Can Bilaterally Activate Nodal Signaling in the Brain Epistatic to Spw Loss of Function

*mbl* embryos frequently exhibit bilateral activation of Nodal pathway genes in the brain despite establishment of normal visceral situs. Axin1 is a critical component of the protein complex that degrades β-catenin (Nusse, 2005), and previous studies have demonstrated that *mbl* mutants have enhanced Wnt signaling activity during late gastrulation in the anterior neural plate (Heisenberg et al., 2001; Houart et al., 2002; Van de Water et al., 2001). This suggests that enhanced Wnt signaling in the prospective epithalamus can lead to activation of Nodal pathway genes in the epithalamus. Supporting this conclusion is the observation that bilateral epithalamic Nodal activation in the absence of effects on the LPM also occurs when LiCl is used to activate Wnt signaling during late gastrulation.

During normal development and in most mutants/morphants that have been examined, Nodal pathway expression in the epithalamus is concordant with expression in the LPM (Halpern et al., 2003). The mechanistic basis for this relationship appears to be the requirement for activity of the Nodal ligand Spw in the LPM to activate Nodal pathway genes in the ipsilateral epithalamus (Long et al., 2003). Given the dependence of epithalamic Nodal signaling upon LPM Nodal signaling, on first analysis, it appears counterintuitive that Wnt pathway activation at late gastrula stage should only affect the epithalamus and not the LPM, whereas Wnt manipulations at later stages concordantly affect both LPM and epithalamus. However, these results are consistent with a model that we have previously proposed in which LPM Nodal signaling is required to ipsilaterally alleviate a repressor of epithalamic Nodal activation that is present on both sides of the brain (Concha et al., 2000). This model helped to explain Nodal pathway expression phenotypes in embryos in which Nodal signaling and/or midline tissue was disrupted. For instance, embryos lacking the activity of the Nodal coreceptor Oep bilaterally express epithalamic Nodal pathway genes, suggesting that Nodal signaling is not obligatory for activation of epithalamic Nodal gene expression.

**Figure 6:**
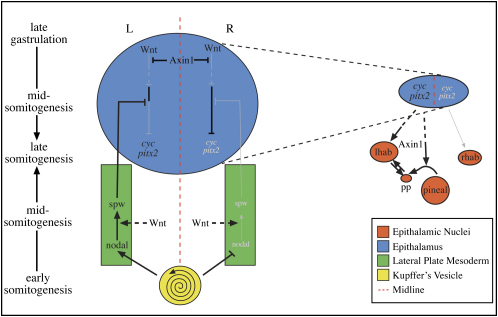
A Model for the Establishment and Elaboration of CNS Asymmetry On the left side of the schematic, events in the epiphysial region of the forebrain from late gastrula to 20 somite stage are indicated in the large blue oval. Events in the LPM are shown in the green boxes. Black lines and lettering indicate that the gene/pathway is active, while gray lines and lettering indicate repression/lack of activation. In the late gastrula neural plate, Axin1 bilaterally represses Wnt signaling in the epithalamus, allowing the establishment of bilateral repression of epithalamic Nodal expression that is carried through to late somite stages. This repression is overcome on the left side by Spw activity from the LPM. In the LPM, the Nodal pathway is activated by Nodal signals emanating from around KV (or the node—see Nakamura et al. [2006]), and activation is subsequently propagated through the left LPM and is shut down on the right. Manipulations to Wnt signaling at this stage (after regression of KV) can disrupt the propagation of Nodal expression. Not shown is the ability of Nodal signals on one side of the LPM to block activation of the pathway on the other side (Ohi and Wright, 2007). Although Spw normally only relieves repression in the left epithalamus, the bilateral symmetry of repression in the epithalamus means that any manipulations or mutations that result in right-sided Spw activity will concordantly lead to right-sided epithalamic activation of Nodal signaling. On the right side of the schematic, events downstream of left-sided activation of Nodal signaling are shown. Nodal signaling influences the laterality of parapineal and habenular asymmetries, but not their establishment per se. How Nodal does this is unknown, and it is not clear whether the pathway exerts its effects primarily through actions on the parapineal, the left habenula, or both (dashed arrows). Axin1 is required for the elaboration of asymmetries downstream of Nodal signaling, both for timely migration of parapineal cells (pp) and for the communication between habenula and parapineal that ensure concordant elaboration of neuroanatomical asymmetries. The spiral symbolises the ciliary flow in KV; lhab, left habenula; rhab, right habenula; L, left; R, right.

Our data indicate that the *mbl* mutation is largely epistatic to Spw loss of function; that is, the *mbl* mutation can lead to activation of Nodal pathway gene expression on both sides of the brain in the absence of Spw activity. We interpret this to imply that the bilateral repression of epithalamic Nodal expression is removed by overactivation of Wnt signaling in the prospective epithalamus, rendering redundant the role of Spw as inhibitor of repression (Figure 6). In this scenario, Axin1 would function in the prospective epithalamus to modulate Wnt signaling and facilitate the establishment of repression. This is consistent with established roles for Axin1 in the regulation of various regulatory genes in the epithalamic region of the anterior neural plate (Heisenberg et al., 2001; Masai et al., 1997; Van de Water et al., 2001). The most parsimonious idea is that the repression established through modulation of Wnt signaling is subsequently alleviated by Spw, but it remains possible that Wnt- and Nodal-dependent mechanisms act in parallel pathways to regulate the repression/activation of Nodal pathway genes in the epithalamus. Indeed, the removal of Spw in *mbl* embryos does reduce the percentage of embryos with epithalamic Nodal activation, suggesting that Spw does retain some activity in the context of enhanced Wnt activity.

To date, no Wnt/Axin1/β-catenin pathway component has been described as being expressed asymmetrically at any time in any species besides Wnt8 in the early chick node (Rodriguez-Esteban et al., 2001) (Figure S3). However, one key feature of our model is that there is no requirement for any asymmetry in the activity of Wnt pathway genes, as repression is required to be bilaterally symmetric. It is the left-sided activity of the Nodal pathway from the LPM at later stages that introduces the asymmetry through removal of repression on the left side of the epithalamus. The right epithalamus remains sensitive to relief from repression, as is evident in situations where Nodal signaling is active in the right LPM, resulting in activation of right-sided epithalamic Nodal signaling. The symmetric activity of Wnt pathway genes in late gastrula neural plate also explains how the *mbl* phenotype can be dissociated from the formation of Kupffer's vesicle (KV) and other events that lead to the establishment of LPM asymmetries (Tabin, 2006).

We speculate that the level, localization, and/or timing of Wnt signaling in the prospective epithalamus establishes a specific level of repression that may be subsequently overcome by unilateral Nodal signaling. This idea is supported by recent data (Inbal et al., 2007 [this issue of *Neuron*]) showing that Six3b/Six7 activity in the neural plate during late gastrulation is required to repress epithalamic Nodal pathway gene expression. Enhanced Wnt signaling, as seen in *mbl* embryos, suppresses Six gene expression (Wilson and Houart, 2004), suggesting that Axin1 activity is required to maintain Six3b/Six7 activity and consequently establish the bilateral repression of epithalamic Nodal pathway gene expression. Furthermore, overexpression of *six* genes can suppress epithalamic Nodal gene expression despite the presence of Spw (Inbal et al., 2007), supporting the idea that specific levels of Wnt and Six activity are required to establish appropriate levels of repression.

Finally, the model provides a likely explanation for an unusual aspect of the *mbl* phenotype. Although epithalamic Nodal signaling is frequently bilateral in *mbl* mutants, the laterality of the brain, as assessed by direction of parapineal migration, is still usually to the left. This is unlike other situations where there is bilateral epithalamic Nodal pathway activation and asymmetries are randomized (Concha et al., 2000; Halpern et al., 2003; Hamada et al., 2002). However, in *mbl* mutants, the left and right sides are not equal. Even if the initial repression is affected to an equal extent on both sides of the neural plate, there is still the activity of left-sided Spw encroaching on the left epithalamus. Thus, if any repression remains within the neural plate of *mbl* mutants, it is likely to be more fully alleviated on the left. This could result in stronger activation of Nodal signaling on the left, even in situations where there is expression of Nodal genes on both sides of the epithalamus. Indeed, using manipulations that lead to bilateral *spw* expression, we found that leftward bias in migration of parapineal cells in *mbl* mutants was reduced (data not shown).

### The Wnt Pathway Influences Nodal Activation in the LPM at Mid-Somite Stages

Activation of Wnt signaling by LiCl in zebrafish or *wnt1* overexpression in medaka during a narrow time window at mid-somitogenesis stages frequently leads to bilateral activation of Nodal pathway gene expression in both LPM and brain. At these stages, key events such as midline development and formation of KV have already occurred. Indeed, KV is beginning to regress at the time at which we manipulate Wnt signaling (Amack and Yost, 2004; Bajoghli et al., 2007; Essner et al., 2005). The manipulations to Wnt signaling at this stage are therefore more likely to directly effect the induction and/or spread of Nodal signals within the LPM than to affect KV per se.

Although the most frequent asymmetry defect following somite stage Wnt manipulations is bilateral activation of Nodal pathway genes, we did also observe reversals with right-sided expression in LPM and brain and absence of left-sided expression. In such situations, either the activation of Nodal signaling on the right leads to abrogation of Nodal expression on the left, or the Wnt pathway independently leads to abrogation of Nodal activation on the left and activation on the right. Although we have not investigated this issue, we favor the idea that activation of Nodal signaling on the right may in some cases result in suppression of the pathway on the left. It is generally believed that the spread of Nodal signaling through the LPM is due to positive regulation of Nodal expression by Nodal activity, and so a localized induction or source of Nodal signals can lead to widespread activation of the pathway in the ipsilateral LPM (Nakamura et al., 2006; Tabin, 2006). Surprisingly, Nodal signaling in the LPM on one side can affect Nodal pathway gene expression in the contralateral LPM, most likely through regulation of Lft genes in midline tissue (Ohi and Wright, 2007; Yamamoto et al., 2003). Thus, it is possible that, in situations where we observed reversed Nodal pathway gene expression, the activation on the right could be responsible for repression on the left.

How might the Wnt pathway be affecting Nodal pathway activation in the LPM? Perhaps Wnt signaling triggers induction of Nodal pathway gene expression, and, once the pathway is activated, then the normal mechanisms of Nodal pathway autoregulation in the LPM propagate a wave of Nodal activation. Given the intrinsic sensitivity of both left and right LPM to the induction and spread of the wave of Nodal activation (Nakamura et al., 2006), one could envisage that a relatively small change in the regulation of Nodal pathway genes could be amplified by autoregulation and result in activation and spread of Nodal signaling through the LPM. Nodal pathway genes can be regulated by Wnt/β-catenin signaling in various contexts (Branford and Yost, 2002; Kioussi et al., 2002). Indeed, in experiments where we mosaically overactivate Wnt signaling, we have observed induction of Nodal pathway genes in both zebrafish and medaka (Figure S4) (Bajoghli et al., 2007).

Following mid-somite stage Wnt pathway manipulations, activation of Nodal pathway gene expression in the epithalamus mirrored the activation in the LPM, consistent with epithalamic activation being dependent upon LPM Spw activity. We have not formally ruled out that these Wnt manipulations might be independently affecting LPM and brain. However, in the rare cases of reversal of LPM Nodal activation, we see concordant epithalamic reversal, suggesting that the two are indeed linked.

### Axin1 Is Required for Development of Concordant CNS Asymmetries Downstream of Epithalamic Nodal Expression

Although the *mbl* mutation frequently results in bilateral activation of epithalamic Nodal signaling, this does not result in concordant randomization of neuroanatomical asymmetries as happens in other situations with bilateral Nodal signaling in the brain (Halpern et al., 2003). For instance, early activation of Wnt signaling by overexpression of *wnt8b* or early treatment with LiCl, both of which result in bilateral Nodal pathway gene expression and similar or more severe morphological phenotypes than in *mbl* embryos, does not prevent the development of habenular asymmetry (Figure S1, Table S2, and data not shown). This implies that Axin1 has additional roles in the development of the lateralized habenulae downstream of Nodal pathway activation. It is well established that Axin1 functions in the Wnt/β-catenin pathway, but, in some circumstances, the protein may be able to influence additional signaling pathways (Kofron et al., 2007; Kusano and Raab-Traub, 2002). The expression of *axin1* in the epithalamus and genes encoding Wnt ligands in and around this region during the period of elaboration of neuroanatomical asymmetries (e.g., Figure S3) is consistent with Axin1 locally modulating Wnt/β-catenin during the elaboration of habenular and parapineal asymmetries. However, as similar phenotypes have not yet been described in any other mutants or following late LiCl treatments (Table S2), the possibility of a noncanonical role for Axin1 in these events remains a possibility.

The habenulae of *mbl* mutants show a severe reduction in the extent of right/left differences, and parapineal cells are delayed in their migration by as much as 2 days. The reduction of *lov* expression in the left habenula coupled with the intermingling of left- and right-sided axon terminals in the IPN in *mbl* embryos is similar to the phenotype observed when the parapineal is ablated (Concha et al., 2003; Gamse et al., 2003; Kuan et al., 2007; I.H.B. and S.W.W., unpublished data). However, as the parapineal is present, in some cases migrates correctly, and usually innervates the habenulae in *mbl* mutants, then *axin1* is the first gene to be implicated in the communication between the parapineal and the left habenula.

An early habenular asymmetry is the precocious generation of neurons on the left (Aizawa et al., 2007), and so one might predict that, in *mbl* mutants, neurogenesis is delayed and habenular cells continue to divide in a manner characteristic of the wild-type right habenula. Indeed, given the ability of Wnt signaling to promote proliferation in many other contexts (Willert and Jones, 2006), one might expect the *mbl* mutant habenulae to show enhanced proliferation more characteristic of the right habenula. However, the formation of habenula precursor cells appears not to be affected in *mbl* mutants as judged by the onset of *cxcr4b* expression, a marker for habenular precursor cells (M. Roussigne and P. Blader, personal communication; data not shown). On the other hand, habenular neurogenesis does appear to be slightly delayed by approximately 2 hr between 32 and 34 hpf as judged by the appearance of HuC+ neurons (data not shown). As described previously (Aizawa et al., 2007), this period is during the time window in which there is a bias to production of early born lateral habenular neurons, and so the slight delay could contribute to the loss of left-sided character. However, it seems unlikely that the delay could completely explain the almost complete lack of left-sided character for *mbl* habenular neurons, as such neurons are generated over a more protracted period of time, extending beyond 34 hpf (Aizawa et al., 2007). The mechanisms by which communication occurs between parapineal and habenular cells are currently not known, and so, although we currently do not know whether the symmetric habenular phenotype of *mbl* embryos is due to Axin1 function in the habenulae, parapineal, or both, we think that the mutant will provide a useful resource for future investigation of such issues.

### Conclusion

The Wnt pathway has previously been implicated in patterning the AP and DV axes, and our study reveals novel roles in left/right patterning. Axin1 activity in the anterior neural plate is required for bilateral repression of Nodal pathway gene expression in the prospective epithalamus. During mid-somitogenesis, Wnt/β-catenin signaling influences restriction of Nodal pathway activation to the left LPM, while downstream of the Nodal pathway, Axin1 is again required for the elaboration of neuroanatomical asymmetries in the brain. The consequence of disruption to Axin1 activity is a lack of concordance of body and brain asymmetries and, uniquely to date, a disruption to the coupling of the habenular and parapineal asymmetries.

## Experimental Procedures

### Fish Line Generation and Maintenance

Zebrafish strains were maintained and bred according to standard procedures (Westerfield, 1995). AB and *tupl* wild-type lines and *masterblind* (*mbl^tm213^*) mutant lines and *mbl^tm213^*xTg(foxD3:GFP) and *mbl^tm213^*xTg(foxD3:GFP); Tg(lft1:GFP) transgenic fish were used. All mutant analyses in this study were performed using transgenic *mbl* mutants derived from an initial cross of *mbl^tm213^* to Tg(foxD3:GFP) transgenic fish, which were kept in an AB background This background resulted in mild *mbl* mutant phenotypes in subsequent generations (Figure 1). The *mbl* mutation abolishes the binding of Axin1 to Gsk3β (Heisenberg et al., 2001), but it is currently unknown whether the mutant Axin1 protein retains any other GSK3β-independent functions.

Embryos and adults of the medaka Cab inbred strain as well as Tg(HS:GFP, HS:wnt1) transgenic fish [formerly named (gfp:HSE:Wnt1)] were used (Bajoghli et al., 2007; Loosli et al., 1998). Stages were determined according to Iwamatsu (Iwamatsu, 1994).

### Cloning and Synthesis of mRNA

To clone *wnt7b* cDNAs, the following degenerated primers were designed: Wnt7b-up (5′-GTGGTVGCYYTGGGHGCVARCATCAT-3′) and Wnt7b-low (5′-CCAKTGGAAYTTRCAGTTRCAYTGCC-3′). RT-PCR reactions were performed using somitogenesis stage zebrafish RNA. The resulting cDNA fragment was cloned into TOPO-TA vector and subcloned into pBluescript, and several clones were sequenced. The obtained sequences showed highest homology with *wnt7b*. As we found slight differences in the sequence and also embryonic expression of two cDNAs (data not shown), we named them *wnt7b* and *wnt7bl* after approval by ZFIN (http://zfin.org/zf_info/nomen.html).

All of the cDNAs used in this work for RNA synthesis were cloned in pCS2+. Capped mRNA was generated using a Message Machine RNA synthesis kit (Ambion) according to the manufacturer's instructions.

### Antibody Labeling and In Situ Hybridization Procedures

Antisense RNA probes were generated using digoxigenin/fluorescein RNA labeling kits (Boehringer-Mannheim). GFP protein was detected by anti-GFP antibody (Torrey Pines Biolabs, TP 401) and acetylated tubulin using antiacetylated tubulin antibody (Sigma T6793). Whole-mount in situ hybridization (Aghaallaei et al., 2005; Bajoghli et al., 2007; Macdonald et al., 1994) and antibody detection were performed with standard procedures (Shanmugalingam et al., 2000). For double whole-mount in situ hybridization BM-Purple (Roche) staining was followed by staining with fast red TR/Naphtol tablets (Sigma) using the manufacturer's protocol. For in situ hybridization analysis coupled with immunohistochemistry, in situ hybridization was performed first.

### Morpholino, mRNA, and cDNA Injection Experiments

The following MOs (Genetools) were used in this study: *tcf3a* Mo (Dorsky et al., [2003]; 2 pmol/embryo) and *spw* Mo (Long et al., [2003]; 10 ng/embryo). In vitro transcribed mRNA was injected at: activated *β-catenin*, 0,6 pg/embryo; *wnt8b,* 6 pg/embryo. DNA (10–20 pg/embryo) encoding GFP:HS:lef-VP16 (Bajoghli et al., 2007) was injected together with the I-SceI meganuclease enzyme as described (Thermes et al., 2003).

### Lithium Chloride and Heat Shock Experiments

LiCl treatments were performed as described (Kim et al., 2002). Embryos younger than tailbud stage were treated for only 15 min. Embryos from tailbud stage onward were dechorionated prior to LiCl treatment.

It is important to note that compounds dissolved in DMSO cannot be used for the study of asymmetry, as DMSO alone disrupts Nodal gene asymmetry even at very low concentrations (<0,1% w/w, data not shown).

Heat shock treatments in zebrafish and medaka were performed at 39°C for 1 and 2 hr, respectively.
