# Supplementary material for: Wnt/Axin1/β-Catenin Signaling Regulates Asymmetric Nodal Activation, Elaboration, and Concordance of CNS Asymmetries
Source: Neuron. 2007 Aug 2;55(3):393–405. doi: 10.1016/j.neuron.2007.07.007 (PMC1940036; doi:10.1016/j.neuron.2007.07.007)
Supplement: Document S1. Supplemental Data [file mmc1.pdf]

**Neuron, Volume *55***

## Supplemental Data

# Wnt/Axin1/β-Catenin Signaling Regulates Asymmetric Nodal Activation, Elaboration, and Concordance of CNS Asymmetries

Matthias Carl, Isaac H. Bianco, Baubak Bajoghli, Narges Aghaallaei, Thomas Czerny, and Stephen W. Wilson

## Supplemental results

### Early activation of Wnt/β-catenin signalling disrupts asymmetric Nodal pathway gene expression

To address if modulation of Wnt signalling can affect asymmetry we activated (exogenous *wnt8b*, *activated-ß-catenin* or *tcf3a* morpholinos) the pathway early in development. A well-described consequence of such manipulations is to disturb AP patterning of the CNS with Wnt signalling posteriorising the rostral brain (Wilson and Houart, 2004; Yamaguchi, 2001). To minimise this effect, we used low amounts of reagents so that we could assess asymmetric structures in embryos in which regionalisation of the brain is relatively normal.

Overexpression of *wnt8b* resulted in concordant bilateral Nodal pathway gene expression in both the LPM and the epithalamus (Figure S1A-B and data not shown). At later stages, embryos showed heterotaxic laterality defects in viscera, heart and brain (Figure S1C-F).

Both the expression of *activated-ß–catenin* and interference with *tcf3a* function induced AP patterning phenotypes similar to those obtained by *wnt8b* overexpression but far fewer embryos exhibited laterality defects (Figure S1A''-F''). Some *tcf3a* morphants showed reversed expression of the habenula marker *lov,* but most other markers were relatively unaffected (Figure S1D'').

### Several Wnt ligands and Wnt pathway genes are expressed in the epithalamus

At least five Wnt ligands are expressed in the epithalamus by the 10 to 14 somite stage (Figure S3) and *wnt16* is expressed in this region soon afterwards (Thisse and Thisse, 2004). Using *flh* expression to define the presumptive pineal complex (Concha et al., 2003; Masai et al., 1997), we find that *wnt1*, *wnt10b* (Lekven et al., 2003; Thisse and Thisse, 2005) and *wnt3a* are expressed caudal and lateral to the presumptive pineal complex (Figure S3A-D), a *wnt7b* homologue (Experimental Procedures) and *wnt8b* are expressed ventral to this domain (Figure S3E-H) and *wnt2* is expressed ubiquitously (Thisse and Thisse, 2004). In addition, several genes that function downstream in the Wnt/ß-catenin pathway are expressed in and/or around the epithalamus at these

stages (Figure S3 and e.g. (Dorsky et al., 2003; Dorsky et al., 1999; Kim et al., 2000; Nasevicius et al., 2000; Veien et al., 2005; Young et al., 2002).

Wnt genes continue to be expressed in the epithalamus after brain asymmetries become morphologically evident with *wnt1* and *wnt7b* expression detectable close to the epiphysis and *wnt3a* and more strongly *wnt8b* expression in the habenulae (Figure S3K-R). At the level of transcription, we did not observe any obvious asymmetries in expression of Wnt pathway components in the brain.

*dkk*, *axin1* and *axin2* are also expressed in and around the epithalamus at somite stages (Figure S3I,J,S,U) and both *axin* genes continue to be expressed in the epithalamus throughout the first few days of development (Figure S3T-V).

**Figure legends for supplementary data:**

**Figure S1. Manipulations that disrupt early Wnt pathway activity result in loss of asymmetry of Nodal pathway gene expression in the LPM and brain and heterotaxia phenotypes**

(A-F) The panels show wild-type expression of genes as indicated on the left. (A'-F') To illustrate the effect of manipulation of Wnt signalling, the panels show the expression of the same genes in *wnt8b* injected embryos. (A,A') Frontal views of the epithalamus and (B,B') dorsal views of 24s stage embryos. Red dotted line indicates the midline, black arrows point at *pitx2* expression in the epithalamus (et) and white arrows at *pitx2* expression in the lateral plate mesoderm (LPM).

(C,C',D,D') Dorsal views of the epithalamus at (C,C') 2 days and (D,D') 4 days of development. (E,E') Dorsal and (F,F') frontal views of 2 days old embryos. The yellow dotted line outlines (E,E') the embryo and (F,F') the heart.

(A''-F'') The graphs illustrate the percentage of embryos with wild-type (WT) left, reversed (rev) right, bilateral (bil), medial (med) or not visible (nv) gene expression upon activation of the Wnt signalling pathway.

lhab, left habenula; pp, parapineal; rhab, right habenula.

**Figure S2. Left and right habenular axon terminals are segregated along the DV axis of the target IPN nucleus**

Dorsal view of the interpeduncular nucleus (IPN) in a 7 days old medaka embryo. DiI/DiD labelling of axonal projections from the habenulae shows the dorso-ventral segregation within the IPN similar to zebrafish (compare with e. g. Figure 4I). dIPN, dorsal and vIPN, ventral IPN.

**Figure S3. Expression of various Wnt pathway genes in and around the epithalamus.**

(A-K,M,O,Q,S-V) Lateral views and (L,N,P,R,T',V') dorsal views focused on the diencephalic epithalamus (et; except S,U). All developmental stages are indicated on the left of each horizontal row of panels and the marker genes used on the top of each vertical row. (A-J) *floating head* (*flh*) expression in the epithalamus (in red) has been used as a landmark. (I,J) *dickkopf* (*dkk*) is expressed in the epithalamic area at mid-somitogenesis stages. Its expression fades shortly later (data not shown). (S,T,T') In zebrafish and Xenopus *axin1* is initially expressed ubiquitously up to tailbud stages but becomes subsequently confined to the fore- and midbrain region (Hedgepeth et al., 1999; Van de Water et al., 2001). (T') Similar to Xenopus we find increased *axin1* expression in the habenula from three days of development onward, but more pronounced on the left side (red dotted circle). (U,V,V') *axin2* is expressed in the dorsal neural tube and CNS including the epithalamus

also at later stages. For further details see supplementary results. ey, eye; hab, habenula (green circle); tc, telencephalon; tec, tectum.

**Figure S4: Mosaic overactivation of Wnt signalling can induce *lft1***

(A,B) Lateral views of the presumptive tail region of tailbud stage embryos, which have been injected with GFP:HS:lef-VP16 DNA (see also Bajoghli et al., 2007), heat shock treated at late gastrulation and 2 hrs later analysed for *lft1* and GFP expression. Ectopic *lft1* expression can be seen in the vicinity of GFP expressing cells.

**References**


Bajoghli, B., Aghaallaei, N., Soroldoni, D., and Czerny, T. (2007). The roles of Groucho/Tle in left–right asymmetry and Kupffer's vesicle organogenesis. Dev Biol *303*, 347-361.

Concha, M. L., Russell, C., Regan, J. C., Tawk, M., Sidi, S., Gilmour, D. T., Kapsimali, M., Sumoy, L., Goldstone, K., Amaya, E., *et al.* (2003). Local tissue interactions across the dorsal midline of the forebrain establish CNS laterality. Neuron *39*, 423-438.

Dorsky, R. I., Itoh, M., Moon, R. T., and Chitnis, A. (2003). Two tcf3 genes cooperate to pattern the zebrafish brain. Development *130*, 1937-1947.

Dorsky, R. I., Snyder, A., Cretekos, C. J., Grunwald, D. J., Geisler, R., Haffter, P., Moon, R. T., and Raible, D. W. (1999). Maternal and embryonic expression of zebrafish lef1. MOD *86*, 147-150.

Hedgepeth, C. M., Deardorff, M. A., and Klein, P. S. (1999). Xenopus axin interacts with glycogen synthase kinase-3 beta and is expressed in the anterior midbrain. MOD *80*, 147-151.

Kim, C. H., Oda, T., Itoh, M., Jiang, D., Artinger, K. B., Chandrasekharappa, S. C., Driever, W., and Chitnis, A. B. (2000). Repressor activity of Headless/Tcf3 is essential for vertebrate head formation. Nature *407*, 913-916.

Lekven, A. C., Buckles, G. R., Kostakis, N., and Moon, R. T. (2003). Wnt1 and wnt10b function redundantly at the zebrafish midbrain-hindbrain boundary. Dev Biol *254*, 172-187.

Masai, I., Heisenberg, C.-P., Barth, K. A., Macdonald, R., Adamek, S., and Wilson, S. W. (1997). *floating head* and *masterblind* regulate neuronal patterning in the roof of the forebrain. Neuron *18*, 43-57.

Nasevicius, A., Hyatt, T. M., Hermanson, S. B., and Ekker, S. C. (2000). Sequence, expression, and location of zebrafish frizzled 10. MOD *92*, 311-314.

Thisse, B., and Thisse, C. (2004). Fast Release Clones: A High Throughput Expression Analysis. ZFIN Direct Data Submission.

Thisse, C., and Thisse, B. (2005). High Throughput Expression Analysis of ZF-Models Consortium Clones. ZFIN Direct Data Submission.

Van de Water, S., Van de Wetering, M., Joore, J., Esseling, J., Blink, R., Clevers, H., and Zivkovic, D. (2001). Ectopic Wnt signal determines the eyeless phenotype of zebrafish masterblind mutant. Development *128*.

Veien, E. S., Grierson, M. J., Saund, R. S., and Dorsky, R. I. (2005). Expression pattern of zebrafish tcf7 suggests unexplored domains of Wnt/beta-catenin activity. Dev Dyn *233*, 233-239.

Wilson, S. W., and Houart, C. (2004). Early steps in the development of the forebrain. Developmental Cell *6*, 167-181.

Yamaguchi, T. P. (2001). Heads or tails: Wnts and anterior-posterior patterning. Curr Biol *11*, R713-724.

Young, R., Reyes, A., and Allende, M. (2002). Expression and splice variant analysis of the zebrafish tcf4 transcription factor. MOD *117*, 269-273.

Table S1: Analysis of asymmetry in *masterblind* (*axin1)* mutant embryos

| marker | wt | reversed | bilateral | not visible | n |
|---|---|---|---|---|---|
| *lft1*-brain | 32 (100) | 0 (0) | 68 (0) | 0 (0) | 82 (62) |
| *pitx2*-brain | 29 (99) | 1 (0) | 70 (1) | 0 (0) | 105 (94) |
| *cyc*-brain | 23 (93) | 3 (1) | 68 (0) | 6 (6) | 31 (88) |
| *otx5*-pp | 38 (100) | 5 (0) | 0 (0)* | 57 (0) | 111 (124) |
| *gfi*-pp | 39 (nd) | 13 (nd) | 36 (nd)* | 12 (nd) | 83 (nd) |
| *cxcr4b*-hab | 85 (86) | 13 (12)** | 2 (2)** | 0 (0) | 53 (50) |
| *zic2a*-hab | 100 (100) | 0 (0)** | 0 (0)** | 0 (0) | 15 (20) |
| *lov*-hab | 5 (96) | 3 (2) | 92 (2) | 0 (0) | 134 (54) |
| *tag1*-hab | 10 (88) | 19 (8) | 71 (4) | 0 (0) | 87 (135) |
| *dex*-hab | 2 (90) | 12 (3) | 86 (7) | 0 (0) | 64 (31) |
| α-acet.tubulin-hab | 2 (95) | 6 (0) | 88 (5) | 4 (0) | 51 (79) |
| DiI/DiD-IPN | 0 (100) | 0 (0) | 96 (0)*** | 4 (0) | 25 (2) |
| *pitx2*-LPM | 100 (nd) | 0 (nd) | 0 (nd) | 0 (nd) | 82 (nd) |
| *spw*-LPM | 94 (96) | 2 (0) | 2 (2) | 2 (3) | 54 (118) |
| *cmlc2*-heart | 84 (95) | 0 (0) | 16 (5)* | 0 (0) | 62 (64) |
| *fkd2*-liver | 98 (100) | 0 (0) | 2 (0)* | 0 (0) | 62 (64) |
| *insulin*-pancreas | 100 (100) | 0 (0) | 0 (0)* | 0 (0) | 62 (64) |

Analysis of *spw* was performed at 16s, *lft1*, *pitx2* and *cyc* was performed at 24s, *cxcr4b*, *otx5*, *gfi*, *zic2a*, *cmlc2*, *fkd2* and *insulin* at 2 days, *tag1* at 3 days and *lov*, *dex*, α-acetylated tubulin and DiI/DiD at 4 days; analysis of *masterblind* siblings is shown in brackets.
*, medial expression instead of bilateral; **, left instead of reversed; right instead of bilateral; ***, ventral instead of bilateral
acet., acetylated; hab, habenula; IPN, interpeduncular nucleus; nd, not determined; n, number of embryos analysed; pp, parapineal; numbers in brackets show values for *masterblind* siblings; all scores shown in column 2-5 show percentages

Table S2. Embryos treated with LiCl show stage dependent disruption of lateralised Nodal pathway gene expression

| stage/marker | wt (left) | reversed (right) | bilateral | not visible | n |
|---|---|---|---|---|---|
| **LiCl-80%** | | | | | |
| *lft1* | 67 | 2 | 31 | 0 | 67 |
| *pitx2*(in LPM) | 80 (100) | 2 (0) | 18 (0) | 0 (0) | 137(52) |
| *otx5* | 30 | 7 | 4* | 59 | 111 |
| *lov* | 89 | 8 | 3 | 0 | 172 |
| **LiCl-90%** | | | | | |
| *lft1* | 91 | 0 | 3 | 6 | 34 |
| *pitx2*(in LPM) | 88 (91) | 5 (8) | 7 (1) | 0 (0) | 86 (89) |
| *otx5* | 78 | 5 | 2* | 15 | 81 |
| *lov* | 94 | 6 | 0 | 0 | 32 |
| **LiCl-tb** | | | | | |
| *lft1* | 95 | 3 | 2 | 0 | 58 |
| *pitx2*(in LPM) | 90 (100) | 5 (0) | 5 (0) | 0 (0) | 38 (40) |
| *otx5* | 94 | 4 | 0 | 2 | 49 |
| *lov* | 94 | 6 | 0 | 0 | 33 |
| **LiCl-12s** | | | | | |
| *lft1* | 69 | 16 | 15 | 0 | 67 |
| *pitx2*(in LPM) | 64 (nd) | 13 (nd) | 23 (nd) | 0 (nd) | 31 (nd) |
| *otx5* | 66 | 19 | 0* | 15 | 90 |
| *lov* | 82 | 13 | 5 | 0 | 93 |
| **LiCl-14s** | | | | | |
| *lft1* | 51 | 20 | 29 | 0 | 99 |
| *pitx2* (in LPM) | 50 (67) | 12 (20) | 38 (13) | 0 (0) | 72 (64) |
| *otx5* | 68 | 19 | 1* | 12 | 131 |
| *lov* | 63 | 32 | 5 | 0 | 85 |
| **LiCl-18s** | | | | | |
| *lft1* | 94 | 1 | 5 | 0 | 108 |
| *pitx2*(in LPM) | 96 (100) | 1 (0) | 3 (0) | 0 (0) | 67 (49) |
| *otx5* | 92 | 3 | 0* | 8 | 149 |
| *lov* | 78 | 17 | 5 | 0 | 218 |
| **LiCl-22s** | | | | | |
| *lft1* | 84 | 3 | 13 | 0 | 46 |
| *pitx2*(in LPM) | 89 (100) | 0 (0) | 11 (0) | 0 (0) | 37 (27) |
| *otx5* | 92 | 5 | 0* | 3 | 60 |
| *lov* | 85 | 14 | 1 | 0 | 94 |
| **LiCl-26hpf** | | | | | |
| *otx5* | 99 | 0 | 1* | 0 | 78 |
| *lov* (30hpf) | 95 (96) | 5 (0) | 0 (4) | 0 (0) | 38 (44) |
| **LiCl-36hpf** | | | | | |
| *otx5* | 100 | 0 | 0* | 0 | 28 |
| *lov* | 100 | 0 | 0 | 0 | 40 |

Embryos were treated with LiCl for 20 min (15 min when earlier than tailbud stage) and fixed at 24s (*lft1*, *pitx2*), 2,5 days (*otx5*) and 4 days (*lov*). *, medial expression instead of bilateral; n, number of embryos scored; nd, not determined
all scores in column 2-5 show percentages

TableS3: Inhibition of GSK-3β with LiCl at 14s results in loss of nodal gene asymmetry

| marker | wt | reversed | bilateral | not visible | n |
|---|---|---|---|---|---|
| *lft1*-brain | 51 | 20 | 29 | 0 | 99 |
| *pitx2*-brain | 50 | 12 | 38 | 0 | 72 |
| *otx5*-pp | 77 | 22 | 0* | 1 | 115 |
| *lov*-habenula | 63 | 32 | 5** | 0 | 85 |
| *pitx2*-LPM | 67 | 20 | 13 | 0 | 64 |
| *cmlc2*-heart | 70 | 18 | 12* | 0 | 89 |
| *fkd2*-liver | 62 | 25 | 13* | 0 | 89 |
| *insulin*-pancreas | 62 | 21 | 17* | 0 | 89 |

Wild-type embryos were LiCl treated at 14s stage for 20 min
All embryos were analysed at 24s (*lft1*, *pitx2*), 2,5 days (*otx5*, *cmlc2*, *fkd2*, *insulin*) and 4 days (*lov*).
*, medial expression instead of bilateral;**, bilateral right instead of bilateral; n, number of embryos analysed; pp, parapineal;
all scores in column 2-5 show percentages

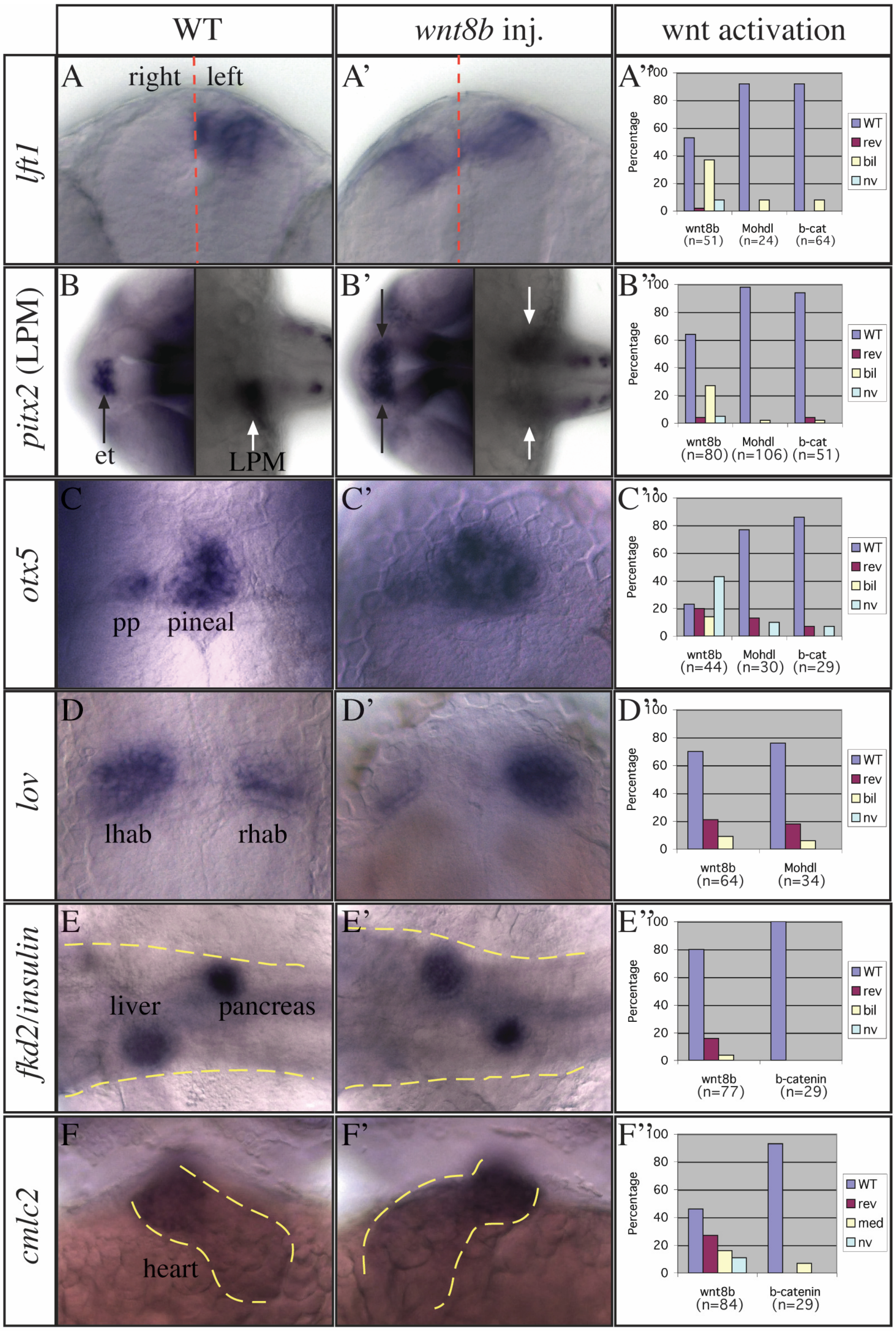


Carl et al., Supplementary Fig. 1

DiI/DiD labelling in medaka

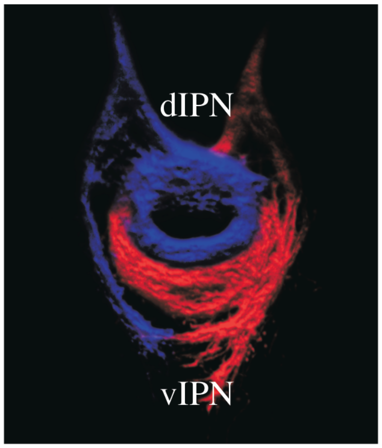


Carl et al., Supplementary Fig. 2

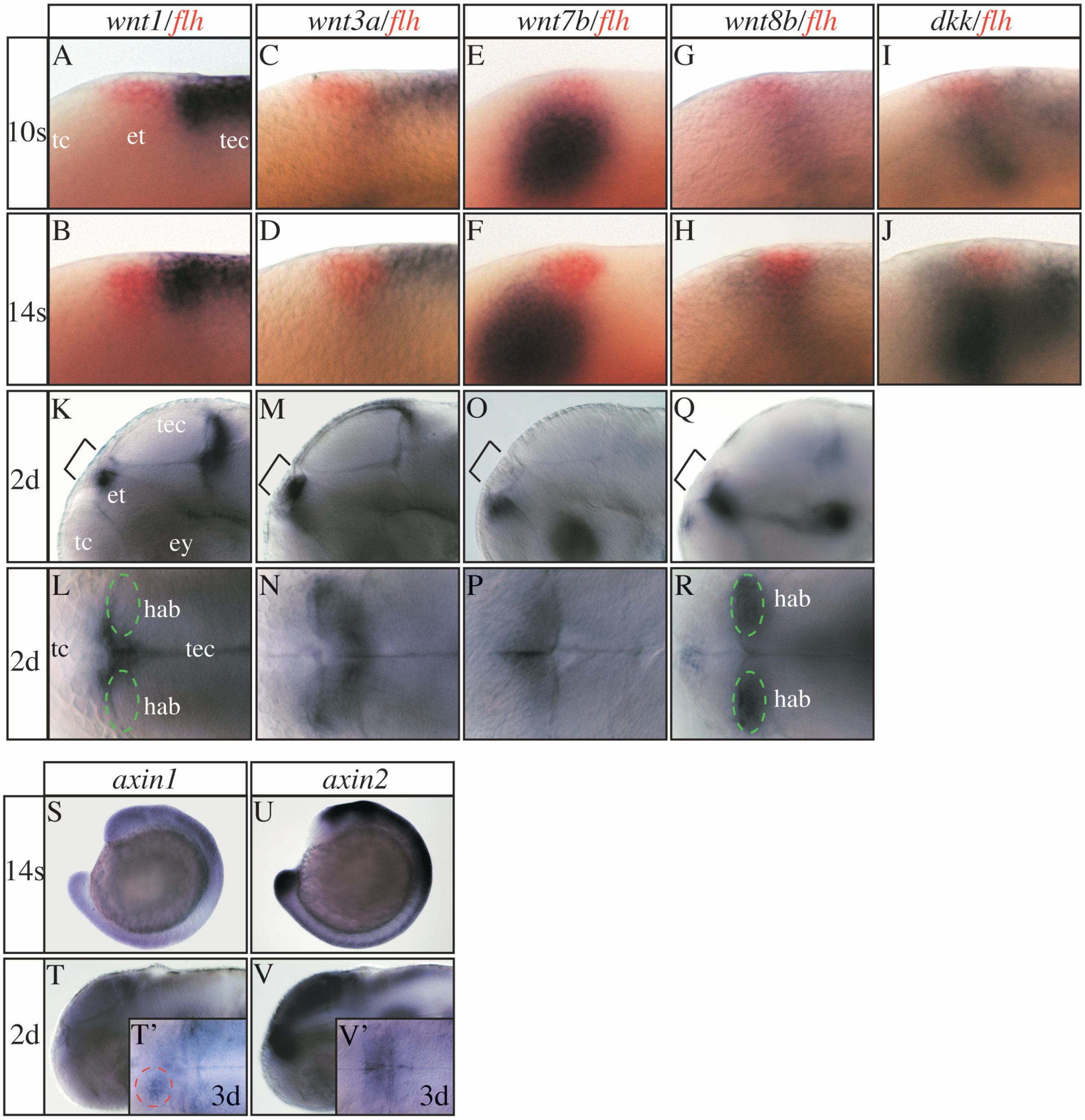


Carl et al., Supplementary Fig. 3

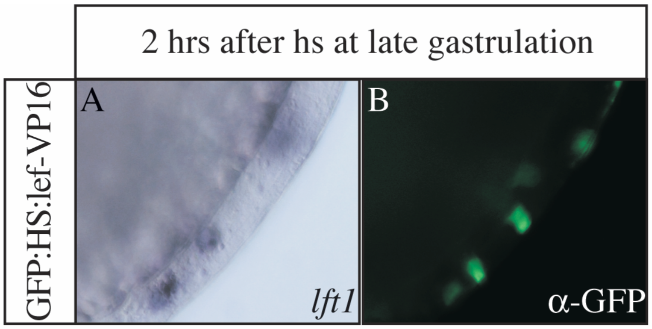


Carl et al., Supplementary Fig. 4
